# Mesoscopic connectome enters the new age of single‐neuron projectome

**DOI:** 10.1002/ctm2.70155

**Published:** 2024-12-30

**Authors:** Ning Li, Hua He, Chun Xu

**Affiliations:** ^1^ Institute of Neuroscience Center for Excellence in Brain Science and Intelligence Technology Chinese Academy of Sciences Shanghai China; ^2^ Department of Neurology Fujian Medical University Union Hospital Fujian Key Laboratory of Molecular Neurology and Institute of Neuroscience Fujian Medical University Fuzhou China; ^3^ Department of Neurosurgery Third Affiliated Hospital of Navy Military Medical University Shanghai China; ^4^ Key Laboratory of Brain Cognition and Brain‐inspired Intelligence Technology Chinese Academy of Sciences Shanghai China; ^5^ University of the Chinese Academy of Sciences Beijing China

**Keywords:** hippocampus, Mesoscopic connectome, single‐neuron projectome

1

The brain area hippocampus has come into the stage centre of neuroscience since the landmark study of Henry Molaison, who lost recent memories and the capability of forming new memories after the extirpation of his medial temporal lobe for epilepsy treatment.^1^ Since then, a growing body of research has elucidated the hippocampal functions in learning and memory,^1^ spatial cognition,^2^ stress responses^3^ and emotional behaviors.^4^ The core circuits within the hippocampal formation comprise a series of unidirectionally connected subregions including dentate gyrus (DG), *cornu ammonis* subfields (CA3 and CA1), and subicular complex.^5^, ^6^ While this basic intrinsic circuity is maintained throughout from the septal (dorsal) to the temporal (ventral) axis in rodents (corresponding to the posterior‐anterior axis in primates), the dorsal and ventral hippocampus (dHC and vHC) have distinct connectivity with cortical and subcortical areas and thereby exhibit disparate physiological functions.^7^, ^8^, ^9^, ^10^

**FIGURE 1:**
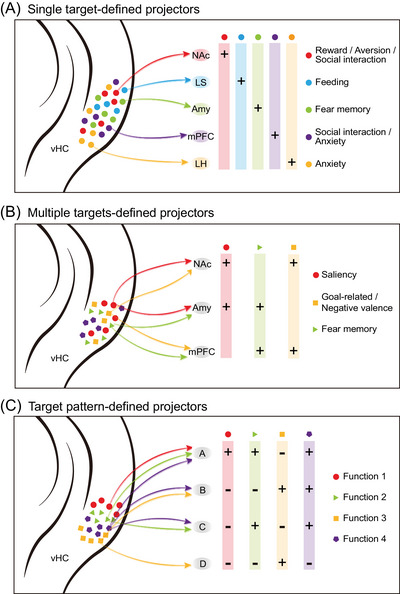
Functional specificity of connectivity‐defined subgroups of hippocampal neurons. (A) Schematic diagram showing functional diversity of ventral hippocampus (vHC) projectors defined by single target areas. Red neurons, nucleus accumbens (NAc) projectors for drug‐related reward,^11^ aversion^22^ and social interaction.^12^ Blue neurons, lateral septum (LS) projectors for feeding.^14^ Green neurons, Amy projectors for fear memory.^13^ Purple neurons, medial prefrontal cortex (mPFC) projectors for anxiety^15^ and social interaction.^16^ Orange neurons, lateral hypothalamus (LH) projectors for anxiety.^18^ (B) Schematic diagram showing functional specificity of vHC projectors defined by simultaneous projections to multiple targets. Red neurons, NAc/Amy projectors for salient signals including reward and aversion.^21^ Orange neurons, NAc/mPFC projectors for goal‐related behaviors.^15^ Green neurons, Amy/mPFC projectors for fear memory.^20^ (C) Schematic diagram showing hypothetical subgroups of vHC neurons that are defined by different target patterns of four brain areas and mediate four types of functions in emotional processing. Red neurons, A‐only projectors for function 1. Green neurons, A/C double projectors for function 2. Orange neurons, B/D double projectors for function 3. Purple neurons, A/B/C triple projectors for function 4.

In rodents, the dHC represents the “cold” hippocampus for spatial cognition and episodic memory, the vHC stands for the “hot” hippocampus for the emotion and stress responses.^7^ The functional diversity of vHC is underscored by its axon projections to various downstream targets, as exemplified by the projection to the nucleus accumbens (NAc) for drug‐induced place preference^11^ and social memory,^12^ the projection to the amygdala (Amy) for contextual fear conditioning,^13^ the projection to lateral septum for feeding,^14^ the projection to medial prefrontal cortex (mPFC) for anxiety,^15^ social interaction^16^ and spatial navigation,^17^ and the projection to lateral hypothalamus for anxiety.^18^ Thus, different subgroups of vHC neurons are defined by their axon projections to individual downstream targets (Figure 1A) and are hypothesized to underlie diverse functions closely related to these targets.^19^ However, this view has been challenged by the fact that many vHC neurons sent co‐projecting axons to multiple brain areas and engaged differently in behavioural functions. For instance, vHC neurons projecting to mPFC and Amy exhibited distinct functions in fear extinction compared to those projecting to either mPFC or Amy.^20^ Ciocchi et al.^15^ showed that vHC neurons projecting to mPFC and NAc were activated by goal‐related behaviours, whereas vHC neurons that had triple projections to mPFC, NAc and Amy were activated by sharp wave/ripples. Furthermore, Chen et al.^21^ reported that vHC neurons projecting to NAc and mPFC showed strong Ca^2+^ signals selectively to negative emotional stimuli, whereas those projecting to NAc and Amy encoded salient signals elicited by both positive and negative stimuli. As depicted in Figure 1B, many studies have demonstrated that the relationship between individual vHC neurons and their downstream targets is not a simple one‐to‐one projection, but one‐to‐multiple targets instead. Thus, the physiological functions of vHC neurons in emotional processing are tightly linked to the target pattern of their downstream brain areas.

The canonical way of circuit tracing by injecting retrograde tracers to one or a few target areas of interest is simply not precise enough, because axon projections to these target areas may or may not have additional collaterals to other target areas (Figure 1A,B). The whole‐brain target areas of single projection neurons have to be taken into account. Thus, the precise definition of the target pattern of projection neurons should consider not only their targeted brain areas but also their non‐targeted areas. Such target pattern‐based definition is particularly important for multi‐collateral projection neurons in many brain areas such as the hippocampus. For instance, the functional diversity of NAc‐projecting vHC neurons, as exemplified by their signalling to multiple emotional stimuli including reward,^11^ aversion^22^ and social interaction,^12^ may have to do with their diversity in axon collaterals to other target areas.^21^ Recent studies on single‐neuron projectomes of the mouse hippocampus have shed light on the whole‐brain target pattern of each neuron and revealed the repertoire of axonal target patterns of hippocampal neurons.^23^ At the whole brain‐wide scale, some subgroups of vHC neurons sent axon projections to only one target, some to only two targets, and some to three or more targets.^23^ These simple and complex axon projections revealed by single‐neuron projectome indicate that the subgroup of vHC neurons defined by retrograde tracing from one target could well be a mixture of vHC neurons projecting to one and multiple targets. As illustrated in Figure 1C, retrograde tracing from target A will label vHC neurons only projecting to A and those projecting to A and C as well as those projecting to A, B and C. The dataset of hippocampal single‐neuron projectomes^23^ offers a guideline to define the target pattern of hippocampal projection neurons. Using intersectional tools,^21^ the A‐only‐projecting neurons could be selectively labelled by excluding A‐projecting neurons with additional collaterals (Figure 1C). We believe that the subgroups of hippocampal neurons delineated by single‐neuron projectome will serve as the circuit basis for diverse and specific functions in emotional processing. The target pattern‐dependent circuit studies would be hallmarks of a new age of mesoscopic connectome with single‐neuron resolution. Functional studies based on single‐neuron projectome data are instrumental for circuit and system neuroscience and potentially provide therapeutic bases for emotional, cognitive and memory‐related disorders, such as depression, dementia and Alzheimer's disease.
